# PulmonDB: a curated lung disease gene expression database

**DOI:** 10.1038/s41598-019-56339-5

**Published:** 2020-01-16

**Authors:** Ana B. Villaseñor-Altamirano, Marco Moretto, Mariel Maldonado, Alejandra Zayas-Del Moral, Adrián Munguía-Reyes, Yair Romero, Jair. S. García-Sotelo, Luis A. Aguilar, Oscar Aldana-Assad, Kristof Engelen, Moisés Selman, Julio Collado-Vides, Yalbi I. Balderas-Martínez, Alejandra Medina-Rivera

**Affiliations:** 1 Laboratorio Internacional de Investigación sobre el Genoma Humano, UNAM, Juriquilla, Mexico; 2 Unit of Computational Biology, Research and Innovation Centre, Fondazione Edmund Mach, 38010 San Michele all’Adige, Italy; 3 Instituto Nacional de Enfermedades Respiratorias Ismael Cosío Villegas, Mexico City, Mexico; 4 Center for Genomic Sciences, UNAM, Cuernavaca, Mexico; 5 Facultad de Ciencias, UNAM, Mexico City, Mexico; 6 Laboratorio Nacional de Visualización Científica Avanzada, LAVIS, UNAM, Juriquilla, Mexico; 7 Department of Biomedical Engineering, Boston University, Boston, Massachusetts USA; 8 CONACYT-Instituto Nacional de Enfermedades Respiratorias Ismael Cosío Villegas, Mexico City, Mexico

**Keywords:** Genetic databases, Chronic obstructive pulmonary disease, Cystic fibrosis

## Abstract

Chronic Obstructive Pulmonary Disease (COPD) and Idiopathic Pulmonary Fibrosis (IPF) have contrasting clinical and pathological characteristics and interesting whole-genome transcriptomic profiles. However, data from public repositories are difficult to reprocess and reanalyze. Here, we present PulmonDB, a web-based database (http://pulmondb.liigh.unam.mx/) and R library that facilitates exploration of gene expression profiles for these diseases by integrating transcriptomic data and curated annotation from different sources. We demonstrated the value of this resource by presenting the expression of already well-known genes of COPD and IPF across multiple experiments and the results of two differential expression analyses in which we successfully identified differences and similarities. With this first version of PulmonDB, we create a new hypothesis and compare the two diseases from a transcriptomics perspective.

## Introduction

A common way to study diseases is by using transcriptomic analysis, which can reveal components of the genome that are active and help us understand which biological processes are affected^1^. Over the years, transcriptomic profiles have been compiled and published in public repositories such as Gene Expression Omnibus (GEO)^2,3^ and ArrayExpress^4^. Having a way to compare transcriptomic data from Chronic Obstructive Pulmonary Disease (COPD) and Idiopathic Pulmonary Fibrosis (IPF) will help to identify common and distinct molecular mechanisms for these two diseases. However, an overwhelming task is to integrate high-throughput data from public repositories, because of platform differences (resulting in batch effects), heterogeneous experimental conditions, and the lack of uniformity on experimental annotations. Wang *et al*. reviewed different approaches in which they discussed tools such as GEO2R^5^, ScanGEO^6^, ImaGEO^7^, BioJupies^8^. These tools reuse public data, reanalyze it consistently, and integrate additional data. Even with these available tools, performing meta-analyses is still challenging^9^. In particular, for COPD and IPF, because the information from only a few experiments is available in these resources, such an analysis requires manual annotation by the user or inclusion of only curated GEO Datasets (also referred as GDS), and only none of them integrates microarray and RNA-Seq data, to our knowledge.

Therefore, we created a curated gene expression lung disease database, PulmonDB, to organize the currently large amount of expression data for both COPD and IPF. To accomplish this task, we used COMMAND > _, a web application previously used to create two successful transcriptomic compendia: one for bacterial genomes, COLOMBOS^10,11^, and the second for grapevine VESPUCCI^12^. While there are other chronic respiratory diseases, such as asthma, cystic fibrosis, and pulmonary hypertension association, among others, given the biological similarities between COPD and IPF, we decided to focus the first version of PulmonDB on these two diseases. We integrated transcriptomic experiments from different sources and their curated annotations, and built an online web resource to facilitate the exploration of gene expression profiles for COPD and IPF creating new hypotheses, and to allow for the identification of co-expression patterns in further analyses.

## Results

**Figure 1:**
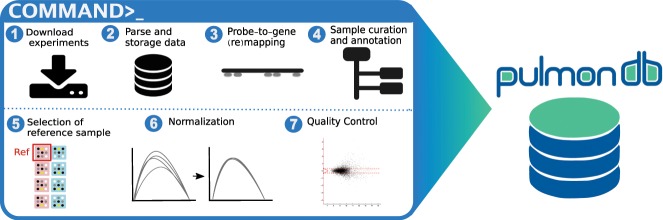
Flow chart of PulmonDB. PulmonDB was created using COMMAND by downloading, parsing and storing COPD and IPF public transcriptomic data into a MySQL database. Then, we remapped microarray probes to establish a uniform gene annotation, and we also created a controlled vocabulary for clinical and biological annotations for each sample. We created contrasts based on the original hypothesis, selecting a sample as the reference. Finally, the data were homogenized and subjected to a quality check.

PulmonDB is a relational database implemented in MySQL with lung disease transcriptome measurements, re-annotated platform probes, and manually curated data with a controlled vocabulary designed for lung diseases (Fig. 1). Tables were created to describe each feature and to connect the information across experiments, samples, measurements, platforms, genes, and annotated information. The full database scheme is provided in Supplementary Fig. 1.

### PulmonDB a curated gene expression lung disease database

PulmonDB is a curated gene expression database of human lung diseases, with RNA-seq and microarray data from different platforms that have been uniformly preprocessed and manually curated to add sample and experiment information. In addition, we developed a website to access and visualize homogenized data (http://pulmondb.liigh.unam.mx/), and we also developed an R package (https://github.com/AnaBVA/pulmondb) to download curated annotation and preprocessed data that can be used for further analysis in the R environment.

**Figure 2:**
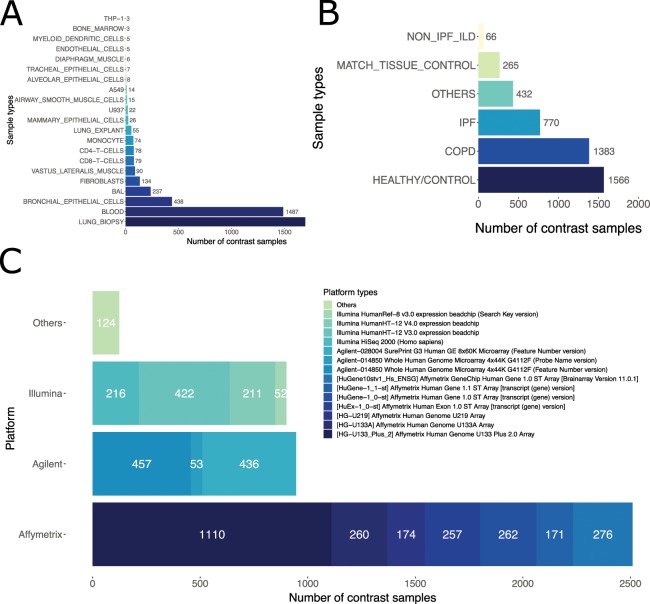
Summary of PulmonDB. (**A**) The number of contrast samples in PulmonDB per biological sample type. (**B**) The number of sample states found in PulmonDB. The color key below the bar chart shows the sectors for COPD patients, healthy/controls, IPF patients, match_tissue_controls (non-cancerous sample from a cancer patient), and other diseases (such as asthma). (**C**) The number of contrast samples measured using each platform (clustered by using Affymetrix, Agilent, Illumina, and other platforms with fewer samples).

Our database has a total of 76 GSEs, corresponding to 4481 unique preprocessed GSM contrasts that used 26 different platforms or GPLs (platform ID from GEO) (Fig. 2C). PulmonDB contains different sample types, we searched for human gene expression experiments related to COPD and IPF without any restriction. Lung biopsies account for 37.8% of samples, and 33.2% are blood samples. However, different cell types can be found in PulmonDB: some of them are primary cells (*e.i*. alveolar macrophages, fibroblasts, alveolar epithelial cells, etc.), and others are cell lines (*e.i*. A549) (Fig. 2A). Of the samples, 34.9% correspond to COPD, 40.5% to control samples (30.9% healthy plus 9.6% match tissue), 17.2% to IPF, and 1.5% to other diseases (Fig. 2B and Supplementary Table 2). We separated control tissues into two groups, “healthy” individuals, as far as the authors are aware and “match_tissue_controls” which refers to tissue samples from a phenotypically healthy region of a patient who had a tumor removed (*e.i*. non-tumor tissue from a cancer patient).

Although other resources reuse and reanalyze GEO data using web interfaces^9^, those tools are not specialized for lung diseases. Their limitations include the need for previous manual curation in each analysis, and they consider a small number of COPD and IPF experiments due to the fact that only curated GEO data are used. We designed a web interface that enables data exploration and visualization to facilitate lung disease analysis. This interface uses Clustergrammer^13^ to visualize gene expression values and the creation of interactive heatmaps that allow data exploration. A valuable feature of Clustergrammer is to be connected to EnrichR^14^, which provides pathway enrichment analysis. All these features together should help to generate new hypotheses about the pathologies of lung diseases to perform exploratory analyses, to visualize specific gene expression across public experiments for comparing results, and to generate new insights based on different data sets.

### PulmonDB can recapitulate gene expression patterns expected in COPD and IPF

**Figure 3:**
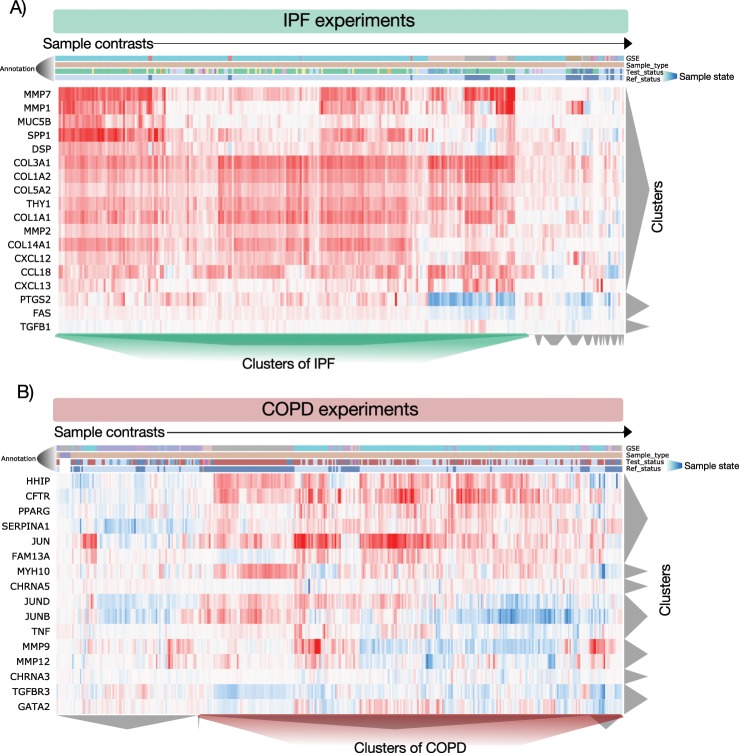
IPF and COPD well-known disease-associated genes. In both heatmaps, rows are genes, and columns are sample contrasts. Both were hierarchically clustered. The first annotation row represents their GSE IDs. The second annotation row is the sample type, LUNG_BIOPSY samples, in light brown. The third and the fourth annotation rows are sample states, the third annotation row represents the test state, and the fourth annotation row is the reference state. (**A**) IPF genes reported being relevant in the literature (CCL18^15^, CXCL12^16^, CXCL13^17^, COL1A1, COL1A2, COL3A1, COL5A2, COL14A1^18^, DSP^19^, FAS^20^, IL-8^21^, MMP1^22^, MMP2^23^, MMP7^22^, MUC5B^19^, SPP1^24^, PTGS2^25^, TGFB1^26^ and THY1^27^). The IPF experiments selected were GSE32537 (pink), GSE21369 (purple), GSE24206 (blue), GSE94060 (grass-green), GSE72073 (lemon yellow), GSE35145 (green), and GSE31934 (yellow). The third and the fourth annotation rows are sample states: light blue, MATCH_TISSUE_CONTROL; dark blue, HEALTHY/CONTROL; turquoise, IPF samples; and grey, NON_IPF_ILD. (**B**) COPD genes reported being relevant in the literature (HHIP^28,29^, CFTR^30,31^, PPARG^32^, SERPINA1^33,34^, JUN^35^, FAM13A^36^, MYH10^35^, CHRNA5^37^, JUND^35^, JUNB^35^, TNF^34^, MMP9^34^, MMP12^34^, CHRNA3^37^, TGFBR3^32^, and GATA2^32^). The COPD experiments selected were GSE27597, GSE37768, GSE57148, GSE8581, and GSE1122. The third and the fourth annotation rows are sample states: light blue, MATCH_TISSUE_CONTROL; dark blue, HEALTHY/CONTROL; red, COPD samples.

To show that PulmonDB can be used to recapitulate previously reported knowledge regarding COPD and IPF biology, we performed a literature search and manually selected relevant genes for each disease. We selected 19 genes related to IPF (not necessarily associated with gene expression in lung tissues) to visualize their gene expression: CCL18^15^, CXCL12^16^, CXCL13^17^, collagens (COL1A1, COL1A2, COL3A1, COL5A2, COL14A1)^18^, DSP^19^, FAS^20^, IL-8^21^, MMP1^22^, MMP2^23^, MMP7^22^, MUC5B^19^, SPP1^24^, PTGS2^25^, TGFB1^26^ and THY1^27^. Then, we selected eight IPF experiments performed with lung tissue biopsy samples (GSE32537, GSE21369, GSE24206, GSE94060, GSE72073, GSE35145, GSE31934), and using the PulmonDB website, we created a heatmap with the gene expression patterns and observed that the hierarchical clustering of these data separates IPF and control data sets (Fig. 3A, green and gray clusters at the bottom). For COPD, we curated 16 genes from the literature that were deemed relevant to this disease: HHIP^28,29^, CFTR^30,31^, PPARG^32^, SERPINA1^33,34^, JUN^35^, FAM13A^36^, MYH10^35^, CHRNA5^37^, JUND^35^, JUNB^35^, TNF^34^, MMP9^34^, MMP12^34^, CHRNA3^37^, TGFBR3^32^, and GATA2^32^. We selected five experiments (GSE27597, GSE37768, GSE57148, GSE8581, GSE1122) performed on lung tissue biopsy samples from COPD patients and controls. Our hierarchical clustering analysis of the expression profiles using the PulmonDB interface allowed us to cluster patients and controls into two different groups (Fig. 3B), similar to the case of IPF. In conclusion, PulmonDB not only helps to recapitulate previously published work (Supplementary Fig. 3) but also helps to verify gene expression stability across experiments. This may help to analyze concordance in different experiments, contrast study results, show implications of using different control groups, etc. We believe this resource can be used to drive, make decisions, and support new hypotheses in experimental laboratories for studying molecular or cellular disease mechanisms.

### Differences and similarities in COPD and IPF

**Figure 4:**
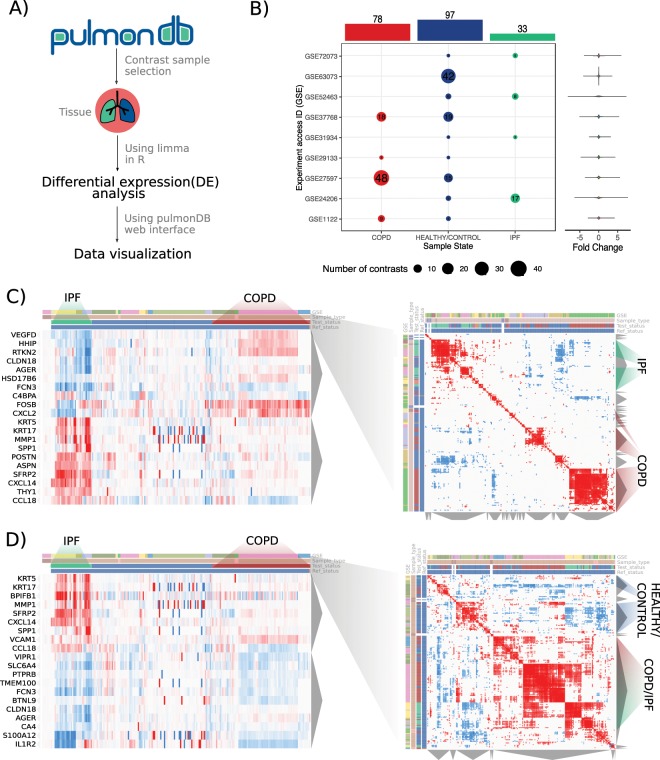
IPF and COPD differentially expressed and similarly expressed genes. (**A**) Flow chart of steps used for COPD and IPF differential expression analysis to evaluate transcriptomic differences and similarities. (**B**) Experiments selected for the analysis, following the criteria of being lung biopsy samples and contrasted with HEALTHY/CONTROL references. The colors represent the sample state: COPD, red; HEALTH/CONTROL, blue; IPF, turquoise. At the top, the bar graph is the total sum of contrasts, rows are the GSE experiments, and each dot is the number of contrasts per experiment from COPD, HEALTHY/CONTROL, or IPF subjects. On the right side, we can see the distributions in violin plots for all sample contrasts per experiment. (**C**) Differentially expressed genes between COPD and IPF. (**D**) Similar genes between COPD and IPF. In both (**C, D**) columns are sample contrasts, rows are genes, the first covariate is colored by each corresponding experiment, the second covariate is the sample type (in this case, lung tissue is shown in light brown), the third row is the test status, and the fourth is the reference status. Columns are ordered by test status and genes by hierarchical clusterization. The right heatmap is the correlation among sample contrasts, and the covariates are the same.

PulmonDB can be used not only to replicate previous knowledge but also to provide a framework to test new hypotheses. In this context, we set out to investigate the differences and similarities between COPD and IPF in lung tissue when compared to samples from healthy individuals (Fig. 4A). Using PulmonDB in the R environment, we selected contrasts where the sample was annotated as lung biopsy and the reference status as HEALTHY/CONTROLs (GSE52463, GSE63073, GSE1122, GSE72073, GSE24206, GSE27597, GSE29133, GSE31934, GSE37768) (Fig. 4B), and then using limma^38^ we assessed differential gene expression between COPD and IPF. We identified 1781 differentially expressed genes (Supplementary Fig. 4). To have a visual representation of the differences between COPD and IPF, we selected the top 20 differentially expressed genes and visualized their expression using the PulmonDB website tool (Fig. 4C). We observed that data sets tend to cluster by test status; Fig. 4C shows IPF contrasts on the left (turquoise), control contrasts in the middle (blue), and COPD contrasts on the right (red). Genes are clustered in two groups (left panel, y-axis); the first gene group (I) is overexpressed in IPF while it is barely expressed or underexpressed in COPD contrasts. By comparison, the second gene cluster (group II) is overexpressed in COPD contrasts and underexpressed in IPF. To correlate similarities among samples, the 20 top differentially expressed genes were used (Fig. 4C, right panel); samples from the same disease group showed higher correlations and tended to have a null or negative correlation with the HEALTHY/CONTROL and the opposite disease (Fig. 4C). For example, FOSB and CXCL2 have opposite behaviors, as both genes are overexpressed in COPD and underexpressed in IPF. FOSB is part of the family of Fos genes that can dimerize with JUN family proteins to form the transcription factor complex AP-1, which is related to COPD^39^. CXCL2 is a chemokine secreted in inflammation that induces chemotaxis in neutrophils^40,41^; these cells are predominant in COPD, and they are key mediators in tissue damage^42^. While neutrophils are also important in IPF, we observed their underexpression in this disease.

We also asked the opposite question, *i.e*., whether we could identify which genes that are shared between these two diseases. We assigned a weight to COPD and IPF expression to perform limma contrasts (Fig. 4D), which enabled us to identify when both diseases drove a differential expression profile. We selected the 20 top differentially expressed genes and visualized their expression patterns using PulmonDB website tool, and we could see that a set of genes was consistently overexpressed or underexpressed in both COPD and IPF. In particular, VCAM1 and FCN3 are differentially expressed in COPD and IPF, with a similar trend in both diseases when compared with HEALTHY/CONTROLs. VCAM1 is the vascular cell adhesion molecule 1, and it is important in the immune response for mediating cellular adhesion in leukocytes^43^; it is overexpressed in these two diseases, suggesting infiltration of immune cells in both pathologies^44,45^. In contrast, FCN3 (or ficolin 3) is underexpressed in both diseases: this gene is a collagen-like protein associated with the innate immune defense, as it activates the lectin complement pathway^46^, which has been shown to be important in pulmonary pathologies^47,48^.

As a result, PulmonDB assisted our analysis of COPD and IPF analogous and antagonist genes and can thus be used to dissect common molecular mechanisms, because both lung diseases are present under heterogeneous conditions with progressive and irreversible phenotypes mainly caused by smoking and by aging, plus both diseases entail cellular matrix remodeling. Furthermore, the differential gene signatures between COPD and IPF might explain the particularities of each disease.

## Discussion

The present methodology had been previously applied for the study of bacterial and grapevine gene expression in different experiments and conditions, allowing for the integration of data from a diverse origin. Here we prove this methodology can also be applied to human data to exploit publicly available resources better, we hope these methods will be taken by other teams to create databases to help understand relevant diseases in other tissues.

PulmonDB can help the scientific community to study which genes have a distinct expression profile in COPD and IPF, explore experiments across technologies and platforms, identify interesting expression patterns across different diseases, generate new hypotheses, and find relationships among clinical or experimental variables. This database also enables comparisons of an updated collection of expression profiles already homogenized for their analyses of specific diseases. Additionally, having different lung diseases (COPD and IPF) in the same database creates the opportunity to observe their similarities and differences. In the future, we aim for PulmonDB to grow and include more diseases. To our knowledge, there is no other resource for transcriptomic analysis focused on the same lung diseases; for this reason, we believe researchers of different backgrounds can use and benefit from the information contained in PulmonDB, by using the web interface and the R package.

An integrated comparable collection of homogenized values with controlled vocabulary describing biological and technical characteristics will facilitate further comparative analyses, such as the study of profiles in COPD and IPF, exploration of experiments across technologies and platforms, identification of interesting coexpression patterns across different diseases, the generation of new hypotheses, and determination of relationships among clinical or experimental variables.

This project sets the foundation to integrate transcriptomics data of other respiratory diseases or related phenotypes and thus facilitates the identification of common and divergent pathways that lead to a pathological state. PulmonDB platform will be expanded in the future to include other lung diseases.

## Methods

### Platform and metadata

Most of the metadata was obtained from GEO. For specific cases, the platform information (.cdf file) was obtained from the Affymetrix website (http://www.affymetrix.com/site/mainPage.affx). Additional information (*e.g*., clinical data, source of the biological sample), was obtained either from metadata or manually curated from the original papers.

### Inclusion criteria for transcriptome data

The experiments currently included in PulmonDB are listed in Supplementary Table 2.

We used two main resources to download raw data and preprocessed counts, GEO and Recount2.

#### Gene expression omnibus

Using GEO^2,3^, we searched datasets related to COPD and IPF for gene expression data. The following queries were used to retrieve the experiments:

(“*pulmonary disease*, *chronic obstructive*”[*MeSH Terms*] *OR COPD*[*All Fields*]) *AND* “*Homo sapiens*”[*porgn*] *AND* (“*gse*”[*Filter*] *AND* (“*Expression profiling by array*”[*Filter*] *OR* “*Expression profiling by high throughput sequencing*”[*Filter*])).

(*Idiopathic pulmonary fibrosis*[*All Fields*] *AND* “*Homo sapiens*”[*porgn*] *AND* (“*gse*”[*Filter*] *AND* (“*Expression profiling by array*”[*Filter*] *OR* “*Expression profiling by high throughput sequencing*”[*Filter*])).

GEO experiments were manually curated, abstracts and related articles were revised, and only datasets confirmed as having COPD and/or IPF samples were considered. In order for an experiment to be included in PulmonDB we used the following criteria: The data set had to be original, samples had to be unique, raw data had to be public and available, platform information must had sequence probes, and custom platforms must have had information to link raw expression signal with the probe sequence. Otherwise, data sets were not taken into account for PulmonDB.

#### Recount2

Recount2 is an online resource with RNA-seq human experiments already preprocessed using Rail-RNA alignment and summarized by gene and exon counts^49^. We used the keywords “IPF” and “COPD” separately in Recount2 to retrieve counts form RNA-seq.

### Compendium creation

The compendium creation process was done as previously described in COLOMBOS and VESPUCCI^10,12^. The platform was developed in bacteria and later employed in grapevine, but in this paper, we used COLOMBOS for the first time in human data. After we selected the datasets using the experiment ID from GEO (GSE), we worked on COMMAND>_^50^.

#### COMMAND

COMMAND stands for COMpendia MANagement Desktop, it is a web application tool that provides a framework to facilitate and perform the following steps: (1) download data from selected experiments, (2) parse files and store data in database form, (3) probe-to-gene (re)mapping process, (4) sample curation and annotation with a controlled vocabulary, (5) selection of references and sample experiments to determine contrasts, (6) homogenization (and normalization) of data, and (7) perform data quality control (Fig. 1). This software can be used for any transcriptomic data^50^.

In more detail, each experiment with a GSE ID, also referred to as a data set, was normalized independently without performing background correction, as explained in^11^. We defined a contrast for each sample with a GSM ID (sample ID from GEO) by using a unique control reference sample per data set. The sample contrast per gene was defined as the log ratio between the expression value in the test condition (*i.e*., IPF, COPD) and the expression value in the reference condition (*i.e*., healthy, untreated, smokers without COPD) (Fig. 1, step 5). This gives every comparison an interpretable biological meaning when combined with extensive manual curated annotation. The condition properties describing the contrasts were then structured in a condition-controlled vocabulary tree. Finally, all contrasts were homogenized, resulting in direct comparable log ratios across all experiments; this information later became part of the final compendium of expression data (Supplementary Fig. 2).

### PulmonDB uses a controlled vocabulary to describe sample metadata

A controlled vocabulary is required to create databases with homogeneous and standard information. For PulmonDB, we created a controlled vocabulary organized in a hierarchical structure that contains terms to annotate transcriptome experiments in lung diseases. We defined classes describing the main categories and terms that can be found in experiments, with some of them as mandatory features (*e.i*. sample type, sample status, and platform). Some non-IPF or non-COPD diseases were included in the controlled vocabulary because the original experiments used them.

Once the controlled vocabulary was established, each article related to the experiment was manually curated, and whenever it was necessary, new terms were added, making the vocabulary flexible and allowing for the inclusion of other diseases to our database in the future. Complete definitions of the terms are provided in Supplementary Table 1.

### Experiment annotation

Each sample was manually annotated using the controlled vocabulary; when necessary, the vocabulary was updated to include new features. The information was curated by experts who reviewed the associated articles and protocols to retrieve data such as age, sex, ancestry, stage of disease or treatment, DLCO (the diffusing capacity of the lung for carbon monoxide, a common functional test), etc., from either GEO or the associated paper.

### Homogenization and quality control

As described before, data homogenization was done with COMMAND>_^11,12^. This step was performed on raw data without background correction, as it has been shown to retrieve more errors^51–53^. A nonlinear model was applied to homogenize raw data. We used RMA Quantile for Affymetrix samples and loess fit for the other platforms. The next step was to summarize probes per transcript using RMA median polish summary from Affymetrix or with data averaged across replicates for the other platforms. After performing the homogenization step, low-quality microarrays were identified using MA plots and histograms of raw and homogenized data.

### Website implementation

PulmonDB has a web interface that uses Clustergrammer (https://clustergrammer.readthedocs.io/index.html)^13^ to visualize gene expression contrasts. Clustergrammer has a frontend in javascript and a backend in python, supporting an interactive web application for gene expression exploration. The PulmonDB web interface requires one or several GSE identifiers and more than two gene names to generate interactive heatmaps.

In addition, Clustergrammer is connected with EnrichR (http://amp.pharm.mssm.edu/Enrichr/)^14^, an integrative web application tool for enrichment analysis that helps the user explore not only potentially differentiated genes but also enriched pathways, facilitating the discovery of transcriptomic signature patterns in lung diseases or related phenotypes.

### COPD and IPF comparative analysis

We used limma 3.40.0 in a Rstudio environment 3.6.0 for our comparative analyses, and the GSE ID was included in the linear model. Then, two contrasts were created: (1) “COPD – IPF”, for obtaining differentially expressed genes between COPD and IPF, and (2) “(COPD + IPF)/2 – CONTROL”, for genes similarly expressed between COPD/IPF and CONTROL. Differential gene expression analyses were adjusted for multiple testing using the false discovery rate (FDR) method, also referred to as Benjamini & Hochberg adjustment. We applied a cutoff of the adjusted *p*-value < 0.05, and after sorting based on the log fold change, the top 20 genes were obtained.

## Supplementary information

Supplementary material

## Data Availability

PulmonDB is accessible (http://pulmondb.liigh.unam.mx/) and through an R package (https://github.com/AnaBVA/pulmondb).
