# Supplementary material for: PulmonDB: a curated lung disease gene expression database
Source: Sci Rep. 2020 Jan 16;10:514. doi: 10.1038/s41598-019-56339-5 (PMC6965635; doi:10.1038/s41598-019-56339-5)
Supplement: Supplementary file 1 — Supplementary material [file 41598_2019_56339_MOESM1_ESM.docx]

**SUPPLEMENTARY DATA**

PulmonDB: a curated lung disease gene expression database

Villaseñor-Altamirano, Ana B.^1^; Moretto, Marco^2^; Maldonado, Mariel^3^; Zayas-Del Moral, Alejandra^4^; Munguía-Reyes, Adrián^3^; Romero, Yair^5^; García-Sotelo, Jair. S.^1^; Aguilar, Luis A^6^; Aldana-Assad, Oscar^1^; Engelen, Kristof^2^; Selman, Moisés^3^; Collado-Vides, Julio^4,7*^; Balderas-Martínez, Yalbi I.^3,8*^; Medina-Rivera, Alejandra^1*^

^1^ Laboratorio Internacional de Investigación sobre el Genoma Humano, UNAM, Juriquilla, Mexico.

^2^ Unit of Computational Biology, Research and Innovation Centre, Fondazione Edmund Mach, 38010 San Michele all’Adige, Italy.

^3^ Instituto Nacional de Enfermedades Respiratorias Ismael Cosío Villegas, Mexico City, Mexico

^4^ Center for Genomic Sciences, UNAM, Cuernavaca, Mexico.

^5^ Facultad de Ciencias, UNAM, Mexico City, Mexico.

^6^ Laboratorio Nacional de Visualización Científica Avanzada, LAVIS, UNAM, Juriquilla, Mexico.

^7^ Department of Biomedical Engineering, Boston University, Boston, Massachusetts, USA

^8^ CONACYT-Instituto Nacional de Enfermedades Respiratorias Ismael Cosío Villegas, Mexico City, Mexico

* To whom correspondence should be addressed to Alejandra Medina-Rivera. Phone: +525556234331, email: [amedina@liigh.unam.mx](mailto:amedina@liigh.unam.mx)

Correspondence should also be addressed to Yalbi I. Balderas-Martínez. Phone:+525554871771, email: [yalbibalderas@gmail.c](mailto:yalbibalderas@gmail.com)

Correspondence should also be addressed to Julio Collado-Vides. Phone: +527773132063, fax: +527773175581, email: [collado@ccg.unam.mx](mailto:collado@ccg.unam.mx)

**Supplementary Figure 1.** Database schema of the MySQL created for PulmonDB. This schema contains the tables with probes, genes, samples, experiments, normalized values, raw values, etc. of PulmonDB, and each line represents the relationship with other tables; they are colored by topic. Experimental information is shown in khaki, homogenized data in purple, condition annotation in lemon green, central ontology structure in pink, probe gene mapping in green, raw data in white, gene information in yellow, ongoing experiment in wheat.


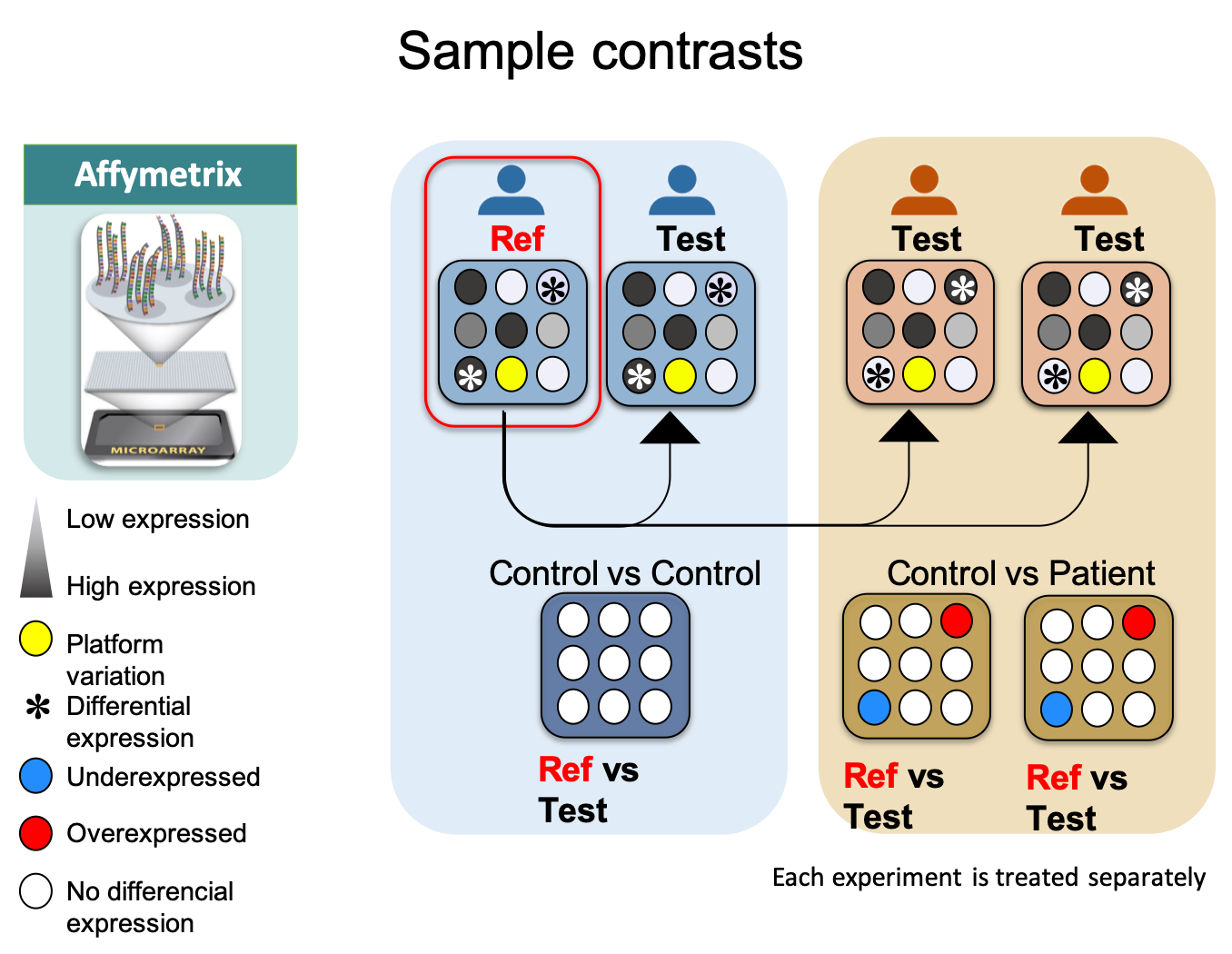


**Supplementary Figure 2**. Sample contrasts, created based on the original hypothesis of each experiment. We used a sample as a reference to make individual contrasts. The contrasts can be between control and control, control vs disease, etc. In this figure, a microarray is represented, with low expression in white and high expression in black, platform variation in yellow, differential gene expression by an asterisk, underexpressed genes in blue, overexpressed genes in red, and genes with no differential expression in white.


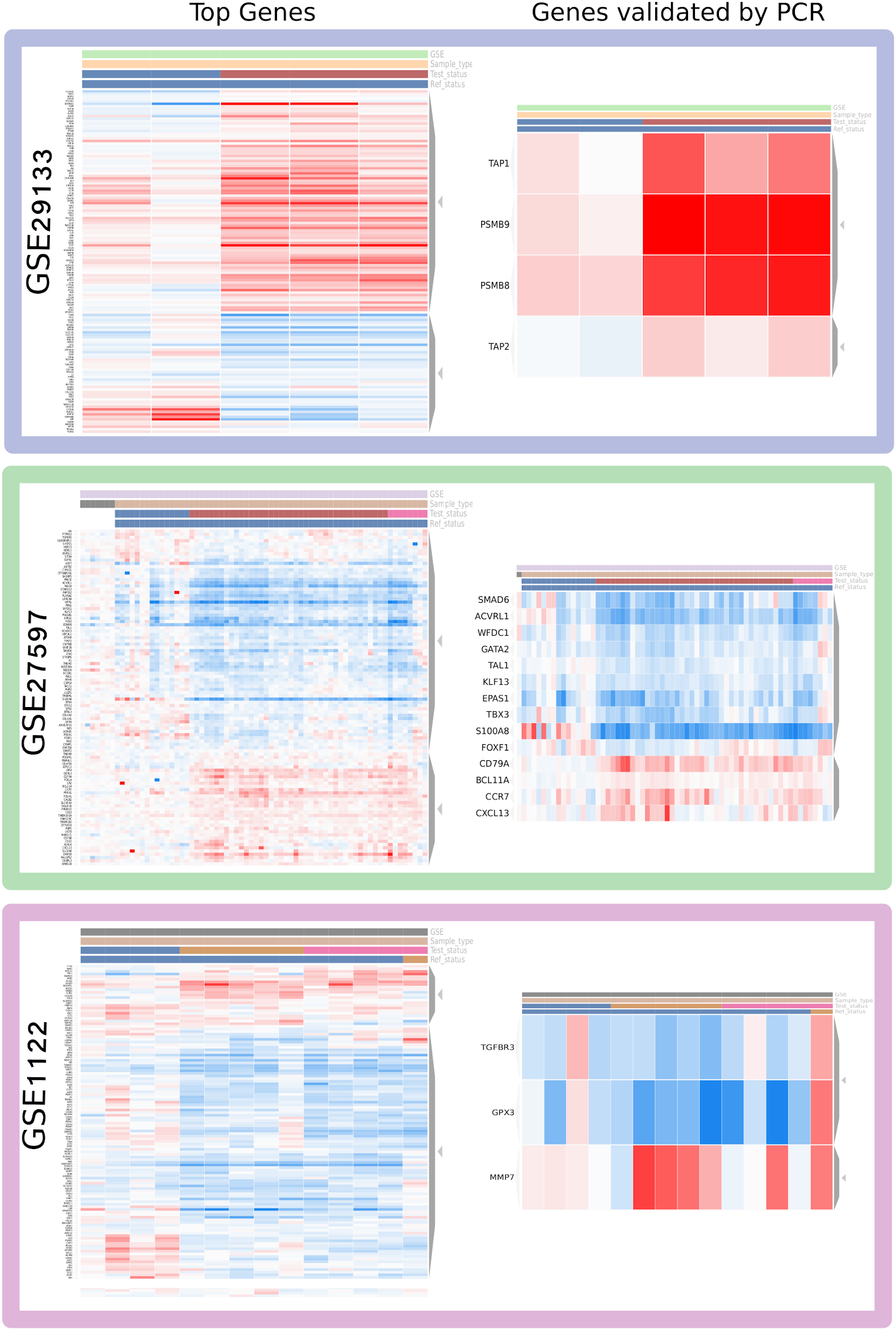


**Supplementary Figure 3.** Top differentially expressed genes in COPD published in three separate papers that also have a related GSE. Purple, genes of Fujino N, *et al*.[^52^](https://paperpile.com/c/5H9CO3/D4W8) using GSE29133; green, genes of Campbell JD, *et al*.[^31^](https://paperpile.com/c/5H9CO3/ZCJs) using GSE27597; pink, genes of Golpon HA, *et al*.[^53^](https://paperpile.com/c/5H9CO3/ZBi3) using GSE1122. The top genes and the genes validated by PCR reported in each article were used as input in PulmonDB website to check reproducibility per experiment in our homogenized contrast values.


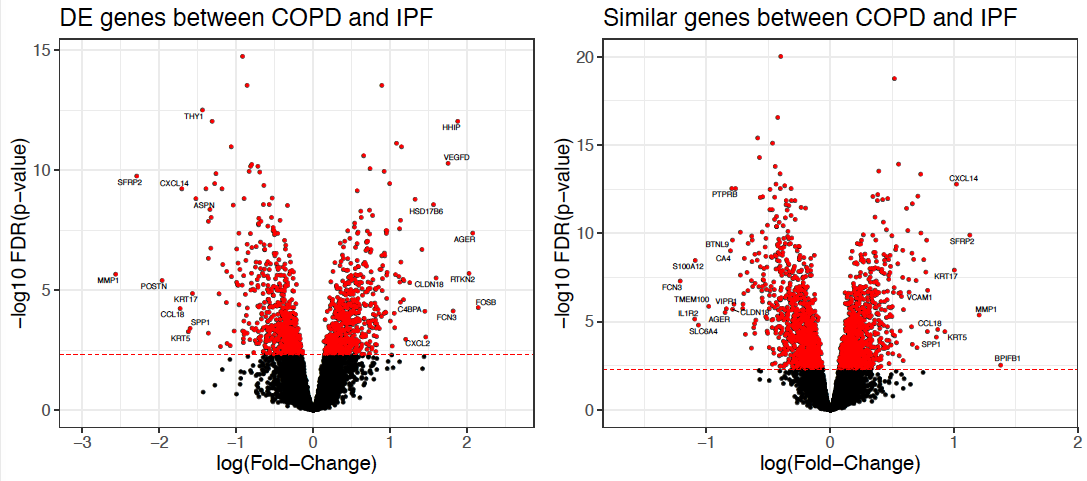


**Supplementary Figure 4.** Volcano plots of differentially expressed genes analyzed for COPD versus IPF and for COPD and IPF compared to a control group. The *x* axis is the log_2_ fold change, the *y* axis is the adjusted *p* -value using FDR in −log_10_, red dots are genes with an adjusted *p*-value of <0.001.

**Supplementary Table 1**. Names and descriptions of the controlled vocabulary used to annotate samples in PulmonDB.

| **Name** | **Description** | **Units** |
| --- | --- | --- |
| **1.** **CLINIC_DETAIL** | | |
| PACK_PER_YEAR | No. of cigarette packs smoked per year | Number of packs per year |
| **1.1. SMOKING_STATUS** | | |
| CURRENT | Current smoker | Boolean |
| FORMER | Former smoker is an ex-smoker individual | Boolean |
| NON_SMOKER | Nonsmoker | Boolean |
| **1.2.**  **STAGE** | | |
| **1.2.1. COPD** | | |
| AE | Acute exacerbation | Boolean |
| GOLD_I | GOLD stage I, mild; FEV1 / FVC < 0.70;FEV1 80% predicted | Boolean |
| GOLD_II | GOLD stage II, moderate; FEV1 / FVC < 0.7050% FEV1 < 80% predicted | Boolean |
| GOLD_III | GOLD stage III, severe;FEV1 / FVC < 0.7030% FEV1 < 50% predicted | Boolean |
| GOLD_IV | GOLD stage IV, very severe;FEV1 / FVC < 0.70FEV1 < 30% predicted or FEV1 < 50% predicted plus chronic respiratory failure | Boolean |
| STABLE | Stable patients | Boolean |
| **1.2.2.** **IPF** | | |
| ADVANCED | IPF advanced stage | Boolean |
| EARLY | IPF early stage | Boolean |
| FAMILIAL_IPF | Familial IPF sample | Boolean |
| SPONTANEOUS_IPF | Spontaneous IPF | Boolean |
| **1.2.2.1 ACUTE_EXACERBATION** | | |
| RAPID | Acute exacerbation – rapid | Boolean |
| SLOW | Acute exacerbation - slow | Boolean |
| **1.3.** **DONOR** | | |
| **1.3.1.** **AGE** | | |
| AGE_PER_INDIVIDUAL | Age of the donor | Years |
| **1.3.1.1. AGE_IN_GROUP** | | |
| SD+/-_AGE | Standard deviation for the group age | Boolean |
| MEAN_AGE | Average age of the group | years |
| RANGE | Group age range | Years |
| **1.3.2.** **ANCESTRY** | | |
| AFRICAN | Ancestry of the donor: African | Boolean |
| ASIAN | Ancestry of the donor: Asian | Boolean |
| CAUCASIAN | Ancestry of donor: Caucasian | Boolean |
| HISPANIC | Ancestry of the donor: Hispanic | Boolean |
| LATINAMERICAN | Ancestry of the donor: Latin American | Boolean |
| NATIVE_AMERICAN | Ancestry of the donor: Native American | Boolean |
| NOT_CAUCASIAN | Ancestry: Not Caucasian | Boolean |
| **1.3.3.** **BODY_MASS_INDEX** | | |
| LOW_BODY_MASS | Low body mass | Boolean |
| NORMAL_BODY_MASS | Normal body mass | Boolean |
| **1.4.** **DISEASE_STATUS** | | |
| AAD | Alpha-1 antitrypsin deficiency-related emphysema | Boolean |
| ASTHMATIC | Asthmatic | Boolean |
| COP | Cryptogenic organizing pneumonia | Boolean |
| COPD | Chronic obstructive pulmonary disease | Boolean |
| CPFE | Combined pulmonary fibrosis and emphysema | Boolean |
| DIP | Desquamative interstitial pneumonia | Boolean |
| EMPHYSEMA | Emphysema | Boolean |
| HEALTHY/CONTROL | Normal status | Boolean |
| IPF | Idiopathic pulmonary fibrosis | Boolean |
| LUNG_CANCER | Donor with lung cancer | Boolean |
| MATCH_TISSUE_CONTROL | Non-cancerous sample from a cancer patient | Boolean |
| NON_IPF_ILD | Interstitial lung disease (ILD) | Boolean |
| NORMAL_RELATIVE | Non affected relative | Boolean |
| NSIP | Nonspecific interstitial pneumonia | Boolean |
| PAH | Pulmonary arterial hypertension | Boolean |
| PH | Pulmonary hypertension | Boolean |
| RB-ILD | Respiratory bronchiolitis | Boolean |
| SARCOIDOSIS | Nodular | self-limiting (N-SL) |
| SS | Systemic sclerosis | Boolean |
| UNCLASSIFIED | Sample with unclassified disease | Boolean |
| **1.5.** **GENDER** | | |
| FEMALE | Female sample | Boolean |
| MALE | Male | Boolean |
| MALE/FEMALE_RATIO | Male/female ratio | Boolean |
| **1.6.** **PHARMACOLOGICAL_TREATMENT** | | |
| STATIN_USER | Statin user | Boolean |
| PHYSICAL_STATUS | NA | NA |
| SEDENTARY | Sedentary | Boolean |
| TRAINED | Trained | Boolean |
| **2.** **FUNCTIONAL_TESTS** | | |
| DLCO (%) | DLCO % | % |
| FEV1/FVC (%) | FEV1/FVC (%) | % |
| FVC (%) | FVC percentage | % |
| **3.** **PROPERTIES** | | |
| PASSAGE (RANGE) | Range of the number of times a cell culture has been subcultured | Boolean |
| PASSAGE (SPECIFIC) | Number of times a cell culture has been subcultured | Boolean |
| **3.1. ANATOMIC_LOCALIZATION** | | |
| LOW_LOBE | Lower lobe | Boolean |
| MIDDLE_LOBE | Middle lobe | Boolean |
| UPPER_LOBE | Upper lobe | Boolean |
| **4.** **SAMPLE** | | |
| **4.1. DIFFERENTIAL_CELL_COUNTS** | | |
| LYMPHOCYTES | Lymphocytes | Boolean |
| MACROPHAGES | Macrophages | Boolean |
| NEUTROPHILS | Neutrophils | Boolean |
| **4.3.** **PROFILE_TYPE** | | |
| MRNA | mRNA | Boolean |
| POLYSOME_ASSOCIATED_RNA | Polysome associated RNA | Boolean |
| SMALL_RNAS | Small RNAs | Boolean |
| TOTAL_RNA | Total RNA | Boolean |
| **4.4.** **SAMPLE_TYPE** | | |
| **4.3.1. CELLS** | | |
| **4.3.1.1. CELL_LINE** | | |
| **4.3.1.1.1. BRONCHIAL_EPITHELIAL_CELLS** | | |
| BEAS-2B | Bronchial epithelial cell line | Boolean |
| **4.3.1.1.2. FIBROBLASTS** | | |
| CCD25Lu | Commercial fibroblast cell line | Boolean |
| HFL1 | HFL1 | Boolean |
| MRC5 | Commercial fibroblast-like cell line MRC5 | Boolean |
| NHLF | Fibroblast commercial cell line | Boolean |
| TIG1 | Fibroblast cell line TIG1 (Tokyo Institute of Gerontology-1) | Boolean |
| TIG7 | Embryonic pulmonary fibroblast cell line TIG7 | Boolean |
| **4.3.1.1.3. MONOCYTES** | | |
| CD14+ | Monocytes CD14+ | Boolean |
| THP-1 | THP-1 cell line | Boolean |
| U937 | Immortalized monocyte cell line | Boolean |
| A549 | Lung carcinoma cell line | Boolean |
| **4.3.1.2. PRIMARY_CULTURE** | | |
| AIRWAY_SMOOTH_MUSCLE_CELLS | Airway smooth muscle cells | Boolean |
| ALVEOLAR_EPITHELIAL_CELLS | Alveolar epithelial cells | Boolean |
| ALVEOLAR_MACROPHAGE | Alveolar macrophage | Boolean |
| BRONCHIAL_EPITHELIAL_CELLS | Bronchial epithelial cells | Boolean |
| CD34+ | CD34+ hematopoietic stem cells | 0,1 |
| ENDOTHELIAL_CELLS | Endothelial cells | Boolean |
| FIBROBLASTS | Fibroblasts | Boolean |
| LARGE_AIRWAY_EPITHELIAL CELLS | Large airway epithelial cells | Boolean |
| LEUKOCYTES | Leukocytes | Boolean |
| MAMMARY EPITHELIAL CELLS | Mammary epithelial cells | Boolean |
| MESENCHYMAL_PROGENITOR_CELL | Mesenchymal progenitor cell | Boolean |
| MONOCYTE | Monocyte | Boolean |
| MYELOID DENDRITIC CELLS | Myeloid dendritic cells | Boolean |
| PBMC | Peripheral blood mononuclear cell | Boolean |
| SMALL_AIRWAYS_EPITHELIAL_CELLS | Small airways epithelial cells | Boolean |
| TRACHEAL EPITHELIAL CELLS | Tracheal epithelial cells | Boolean |
| **4.3.1.2.1. T-CELLS** | | |
| CD4-T-CELLS | CD4 T cells | Boolean |
| CD8-T-CELLS | CD8 T cells | Boolean |
| IL-6R_CD8 | IL-6R CD8 T cells | Boolean |
| IL-7R_CD8 | IL-7R CD8 T cells | Boolean |
| **4.3.1.2.1.1. DIFFERENTIATION_STATUS** | | |
| EFFECTOR | Differential status of T cells | 0,1 |
| MEMORY | Differentiation status of T cells | 0,1 |
| NAIVE | Differentiation status of T cells | 0,1 |
| **4.4.1.** **FLUID** | | |
| BAL | Bronchoalveolar lavage (BAL) | Boolean |
| BLOOD | Blood (sample type) | Boolean |
| SALIVA | Saliva | Boolean |
| SERUM | Serum | Boolean |
| SPUTUM | Sputum | Boolean |
| **4.4.2.** **TISSUE** | | |
| BONE_MARROW | Hematopoietic stem cells | Boolean |
| BONE_MARROW | Hematopoietic stem cells | Boolean |
| DIAPHRAGM_MUSCLE | Diaphragm muscle | Boolean |
| LUNG_BIOPSY | Lung biopsy | Boolean |
| LUNG_EXPLANT | Sample taken from a lung explant | Boolean |
| VASTUS_LATERALIS_MUSCLE | Vastus lateralis muscle | Boolean |
| REFERENCE RNA | Stratagene Universal Human Reference RNA (catalog number 740000) | Boolean |
| **4.5.** **TREATMENT** | | |
| ADENOVIRUS_TREATMENT | Cell culture treated with adenovirus to activate green fluorescent protein (GFP) | Boolean |
| AIR_TREATMENT | Air treatment | Boolean |
| ALI_CULTURE | Air-liquid interface culture | Days |
| ANTI_CD3_AND_ANTI_CD28 | Anti-CD3/CD28 cell activator | Boolean |
| ATRA | All-trans retinoic acid concentration | mM |
| AZA | 5'-Azacytidine | uM |
| CD3/28 | Anti-CD3/CD28 beads | Boolean |
| CIGARETTES | Number of cigarettes per year | Number_of_Cigarettes |
| CONTRACTILE_ECM | Contractile extracellular matrix | Boolean |
| CONTROL_ECM | Control extracellular matrix | Boolean |
| CULTURED | Cultured samples | Boolean |
| CURCUMIN | Curcumin | uM |
| EBV | Epstein-Barr virus infection | |
| FLUTICASONE | Corticosteroid treatment for COPD | Boolean |
| FLUTICASONE/SALMETEROL | Corticosteroid treatment for COPD | Boolean |
| FLUTICASONE_6MONTHS/PLACEBO_24MONTHS | Treatment for COPD | Boolean |
| GHK | Glycyl-L-histidyl-L-lysine peptide (nano molar concentration) | nM |
| HOMOZYGOUS_NULL | Homozygous null gene | null_gene |
| IFN_GAMMA | Interferon gamma concentration | U/mL |
| IL-13 | IL-13 | ng/mL |
| IPF_ECM | Fibrotic extracellular matrix | Boolean |
| LIA | Leukocyte-induced angiogenesis assay | Boolean |
| NON_CONTRACTILE_ECM | Non contractile extracellular matrix | Boolean |
| NON_CULTURED | Noncultured | Boolean |
| NTHi375 | H. influenzae strain NTHi375 | CFU/mL |
| OXIDATIVE_STRESS | H2O2 concentration (micromolar) | μM |
| OXYGEN_TREATMENT | Oxygen treatment | Boolean |
| P3C | Pam3Cys | μg/mL |
| PGE2 | Prostaglandin E2 | μg /mL |
| PLACEBO | Placebo | Boolean |
| SLPS | Standard lipopolysaccharide (LPS) | ug/mL |
| SMOKE_EXPOSURE | Treatment with smoke exposure | Boolean |
| TGF_BETA | Exogenous TGF-beta levels | ng/mL |
| TNF_ALFA | TNF-alpha concentration | ng/mL |
| TRAINING_EXERCISE | Training exercise (in weeks) | Number of weeks |
| TRANSFECTED | Transfection with the gene (fill in the blank with the gene name) | Gene |
| UNTREATED | No treated cells/tissue | Boolean |
| UPLPS | Ultrapure Escherichia coli O111:B4 LPS | μg/mL |
| **4.4.1. TIME_OF_STIMULI** | | |
| HOURS | HOURS | HOURS |
| MONTHS | MONTHS | MONTHS |
| WEEKS | WEEKS | WEEKS |
| **4.6.** **TYPE** | | |
| GROUP | Number of individuals that comprise the pool or group | individuals |
| INDIVIDUAL | If the donor is an individual | Boolean |
| **5.** **STUDIED_DISEASE** | | |
| COPD_EXPERIMENT | Used to classify the disease studied in the experiment. NOTE: It does not describe whether donor is healthy or diseased. | Boolean |
| CPFE_EXPERIMENT | Combined pulmonary fibrosis and emphysema; used to classify the disease studied in the experiment. | Boolean |
| IPF_EXPERIMENT | Used to classify the disease studied in the experiment. NOTE: It does not describe whether donor is healthy or diseased. | Boolean |

**Supplementary Table 2**. GEO Series available in PulmonDB with the number of sample contrasts created per experiment.

| GEO Series ID | Lung disease | Experiment original name (as published) | Pubmed ID related | Platform(s) | Sample contrasts |
| --- | --- | --- | --- | --- | --- |
| GSE10038 | COPD | Upregulation of expression of matrix metalloproteinases in alveolar macrophages of HIV-1+ smokers with early emphysema | 19605697 | HG-U133_Plus_2 | 10 |
| GSE107426 | COPD | Results of differentially expressed lncRNAs in COPD and healthy smokers using the Arraystar Human LncRNA microarray | NA | GPL16956 | 1 |
| GSE10896 | COPD | Impact of curcumin on human monocytes (U937 cells) exposed to oxidative stress | 18421014 | HG-U133_Plus_2 | 22 |
| GSE1122 | COPD | Emphysema lung tissue gene expression profiling | 15284076 | GPL80 | 14 |
| GSE12815 | COPD | PI3K pathway activity in the normal airway of smokers with lung cancer, and in smokers with airway dysplasia | 20375364 | GPL5175,GPL96, HG-U133_Plus_2 | 67 |
| GSE13896 | COPD | Smoking-dependent reprogramming of alveolar macrophage polarization: implication for pathogenesis of COPD | 19635926 | HG-U133_Plus_2 | 69 |
| GSE1650 | COPD | COPD study | 15374838 | GPL96 | 29 |
| GSE16972 | COPD | COPD-specific gene expression signatures of alveolar macrophages as well as peripheral blood monocytes overlap and correlate with lung function | 21430361 | GPL96 | 11 |
| GSE2125 | COPD | Isolated alveolar macrophages | 16166618 | HG-U133_Plus_2 | 44 |
| GSE23704 | COPD | Gene expression profiling on bronchoalveolar lavage (BAL) cells treated with all-*trans*-retinoic acid (ATRA) | 22204820 | HG-U133_Plus_2 | 1 |
| GSE26296 | COPD | Genome-wide analysis of lung myeloid dendritic cells gene expression from healthy or emphysema subjects | 22261033 | GPL6884 | 5 |
| GSE27536 | COPD | Vastus lateralis biopsies from healthy and COPD patients before and after 8 weeks of exercise training | 21909251 | HG-U133_Plus_2 | 53 |
| GSE27543 | COPD | Vastus lateralis biopsies from healthy and COPD patients before and after 3 weeks of endurance training | 21909251 | GPL201 | 15 |
| GSE27597 | COPD | A gene expression signature of emphysema-related lung destruction and its reversal by the tripeptide GHK | 22937864, 24380442, 24380443, 24089408 | GPL13243 | 70 |
| GSE29133 | COPD | Transcriptome in alveolar epithelial type II cells isolated from normal and COPD lungs of adult human | 23117565 | HG-U133_Plus_2 | 5 |
| GSE30027 | COPD | Human lung CD8+ T cells compared with paired non-naive peripheral blood CD8+ T cells | NA | GPL6947 | 11 |
| GSE33337 | COPD | miRNA changes in mild and moderate emphysema correlate with target gene expression in vivo and in vitro [target gene expression data] | 24479666 | GPL6947 | 6 |
| GSE34562 | COPD | IL-6R identifies early differentiated human effector memory CD8+ T cells that potently expand and produce IL-2 and IL-13 | 25390970 | GPL10558 | 5 |
| GSE37147 | COPD | Bronchial airway gene expression reflects a COPD-associated field of injury that changes with disease severity and is reversible with therapy | 23471465 | GPL13243 | 269 |
| GSE37693 | COPD | Gene expression effects of IL-13 on primary human airway epithelial cells | 23187130 | GPL6947 | 7 |
| GSE37768 | COPD | Expression data in lung tissue from moderate COPD patients, healthy smokers and nonsmokers | NA | HG-U133_Plus_2 | 37 |
| GSE40885 | COPD | Data expression in alveolar macrophages induced by lipopolysaccharide in humans | 22952057 | HG-U133_Plus_2 | 13 |
| GSE45251 | COPD | The inflammatory response of human airway smooth muscle cells | 23590298 | GPL6480 | 15 |
| GSE46903 | COPD | Transcriptome-based network analysis reveals a spectrum model of human macrophage activation [Expression] | 24530056 | GPL6947 | 383 |
| GSE47460 | COPD | Gene expression profiling of chronic lung disease for the Lung Genomics Research Consortium | 29988126, 27609773, 27609774 | GPL5175, GPL13607,GPL4133 | 581 |
| GSE475 | COPD | Chronic obstructive pulmonary disease | NA | GPL96 | 6 |
| GSE47718 | COPD | Smoking dysregulates the human airway basal cell transcriptome at COPD-linked risk locus 19q13.2 | 24498427 | GPL11154 | 16 |
| GSE47929 | COPD | Gene expression profiles of Siglec-14/THP-1 and Siglec-5/THP cell lines, with or without NTHi stimulation | 24994897 | HG-U133_Plus_2 | 3 |
| GSE55962 | COPD | Systemic inflammatory response to smoking in chronic obstructive pulmonary disease: evidence of a gender effect | 24830457 | GPL13667 | 105 |
| GSE56341 | COPD | Gene expression profiles of COPD and nonCOPD small airway epithelia | 24298892 | GPL6244 | 21 |
| GSE56768 | COPD | Whole blood and isolated blood cell transcriptomics in COPD | NA | HG-U133_Plus_2 | 431 |
| GSE56768 | COPD | Whole blood and isolated blood cell transcriptomics in COPD | NA | HG-U133_Plus_2 |  |
| GSE57148 | COPD | Characterizing gene expression in lung tissue of COPD subjects using RNA-seq | 25834810, 29871630 | GPL11154 | 186 |
| GSE60399 | COPD | Expression analysis of stable chronic obstructive pulmonary disease and acute exacerbation of chronic obstructive pulmonary disease | 25407108 | GPL18451 | 4 |
| GSE62974 | COPD | RNA sequencing (RNA-SEQ) of EPAS1 knockdown by siRNA in endothelial cells | 25569234 | GPL16791 | 5 |
| GSE63073 | COPD | Genes related to emphysema are enriched for ubiquitination pathways | 25432663 | GPL3991 | 42 |
| GSE69134 | COPD | Genome expression profiling identifies host-directed antimicrobial drugs against respiratory infection by nontypable *Haemophilus influenzae* | 26416856 | GPL887 | 7 |
| GSE69557 | COPD | CD34^+^ DNAM^bright^ CXCR4^+^ CLP mobilized in chronic inflammation | 26436997 | GPL6244 | 3 |
| GSE69818 | COPD | COPD lung tissue expression | 26735770 | GPL13667 | 69 |
| GSE7557 | COPD | Human bronchial epithelial cells_Passage 3 vs. passage 0 | 17965775 | GPL5102 | 3 |
| GSE76705 | COPD | Complex disease subtypes identified by network-based clustering of gene expression data: application to COPD | 26773458 | HG-U133_Plus_2 | 228 |
| GSE77344 | COPD | Gene expression in chronic obstructive pulmonary disease (COPD) patients | http://dx.doi.org/10.1101/038794 | GPL11532 | 171 |
| GSE81614 | COPD | Novel RNA-binding activity of NQO1 promotes SERPINA1 mRNA translation | 27515817 | GPL10558 | 14 |
| GSE8581 | COPD | Human chronic obstructive pulmonary disorder (COPD) biomarker | 18849563 | HG-U133_Plus_2 | 58 |
| GSE8608 | COPD | MDM from COPD patients and healthy subjects after treatment with LPS or fine and ultrafine particles | 18084737 | HG-U133_Plus_2 | 5 |
| GSE87098 | COPD | Expression data of mucociliated human airway epithelia on-chip with or without exposure to whole cigarette smoke under physiological breathing | 27894999 | GPL16686 | 14 |
| GSE994 | COPD | Effects of cigarette smoke on the human airway epithelial cell transcriptome | 15210990 | GPL96 | 74 |
| GSE1786 | Lung function in elderly | Vastus lateralis biopsies from healthy trained and sedentary males | 16260967 | GPL96 | 22 |
| GSE101286 | IPF | Gene expression profiling of idiopathic interstitial pneumonias (IIPs): identification of potential diagnostic markers and therapeutic targets | 28821283 | GPL6947 | 14 |
| GSE11196 | IPF | Fibrotic myofibroblasts manifest genome-wide derangements of translational control | 18795102 | HG-U133_Plus_2 | 44 |
| GSE15197 | IPF | RNA expression profiling of lung tissue identifies mutually distinct molecular signatures in PAH and PH secondary to IPF | 20081107 | GPL6480 | 38 |
| GSE19976 | IPF | Gene expression analysis of lung biopsies from patients with two different forms of pulmonary sarcoidosis | 20194811 | GPL6244 | 14 |
| GSE21369 | IPF | Gene expression profiles of interstitial lung disease (ILD) patients | 21241464 | HG-U133_Plus_2 | 28 |
| GSE24206 | IPF | Validated gene expression signatures of idiopathic pulmonary fibrosis | 21974901 | HG-U133_Plus_2 | 22 |
| GSE26594 | IPF | Increased cell surface Fas expression is necessary to sensitize lung fibroblasts to Fas ligation-induced apoptosis: implications for fibroblast accumulation in idiopathic pulmonary fibrosis | 21632719 | HG-U133_Plus_2 | 5 |
| GSE28221 | IPF | Peripheral blood mononuclear cell gene expression profiles may predict poor outcome in idiopathic pulmonary fibrosis | 24089408 | GPL4133,GPL5175 | 136 |
| GSE31934 | IPF | To examine the expression of Sulf1 and Sulf2, as well as other glycan-related genes, in human Idiopathic pulmonary fibrosis (IPF) lungs compared to normal lung samples | NA | GPL11097 | 5 |
| GSE32537 | IPF | Molecular phenotyping of the idiopathic interstitial pneumonias [mRNA] | 23783374 | GPL6244 | 216 |
| GSE33566 | IPF | The peripheral blood transcriptome predicts the presence and extent of disease in idiopathic pulmonary fibrosis | 22761659 | GPL4133 | 122 |
| GSE35145 | IPF | Gene expression changes in IPF | 22700861 | GPL10558 | 7 |
| GSE38958 | IPF | Profiling of gene expression in idiopathic pulmonary fibrosis | 26286721 | GPL5175 | 114 |
| GSE44723 | IPF | Bleomycin induces molecular changes directly relevant to idiopathic pulmonary fibrosis: A model for “active” disease | 23565148 | HG-U133_Plus_2 | 12 |
| GSE45686 | IPF | An extracellular matrix-driven positive feedback loop regulates translation in idiopathic pulmonary fibrosis | 24590289 | GPL10558 | 38 |
| GSE48149 | IPF | Lung tissues in systemic sclerosis have gene expression patterns unique to pulmonary fibrosis and pulmonary hypertension | 21360508 | GPL16221 | 52 |
| GSE49072 | IPF | Alveolar macrophage gene expression in human pulmonary fibrosis | 23924348 | GPL96 | 83 |
| GSE52463 | IPF | Transcriptome analysis reveals differential splicing events in IPF lung tissue | 24647608 | GPL11154 | 14 |
| GSE52612 | IPF | Forkhead Box F1 (FOXF1) represses fibroblast functions relevant to fibrogenesis | 25260753 | GPL13607 | 7 |
| GSE53845 | IPF | 40 IPF patients and 8 healthy controls | 25217476 | GPL4133 | 94 |
| GSE5457 | IPF | Retinoic acids exposure alters TGF-beta1-induced epithelial mesenchymal transition via Wnt5b expression | 18621908 | GPL96 | 7 |
| GSE6804 | IPF | Expression data of myofibroblast isolated from patients with idiopathic pulmonary fibrosis | 17986007 | GPL201 | 2 |
| GSE69764 | IPF | Identification of the gene expression in IPF lung fibroblasts after demethylation | 26442443 | HG-U133_Plus_2 | 5 |
| GSE71351 | IPF | Global gene expression profiles of fibroblasts from the lungs of patients with idiopathic pulmonary fibrosis: The role of CCL8 | 28057004 | GPL10558 | 11 |
| GSE72073 | IPF | Expression data from lung tissues of IPF patients and normal controls | 26453058 | GPL17586 | 7 |
| GSE73854 | IPF | Developmental programming in idiopathic pulmonary fibrosis (IPF) | 27869174 | HG-U133_Plus_2 | 7 |
| GSE94060 | IPF | Human lung MPC | 28463231 | GPL6244 | 8 |
| GSE38934 | IPF/COPD | Gene expression profiling of lung tissues from patients with combined pulmonary fibrosis and emphysema | 23025845 | HG-U133_Plus_2 | 5 |

**Supplementary Table 3**. A full extended table with number of contrasts per disease type.

| Disease type | Number of contrasts |
| --- | --- |
| ASTHMATIC | 15 |
| COPD | 1383 |
| EMPHYSEMA | 26 |
| HEALTHY/CONTROL | 1566 |
| IPF | 770 |
| MATCH_TISSUE_CONTROL | 265 |
| NON_IPF_ILD | 66 |
| NORMAL_RELATIVE | 24 |
| PAH | 26 |
| SARCOIDOSIS | 7 |
| UNCLASSIFIED | 30 |

**Supplementary Table 4**. A full extended table with number of contrasts per sample type previously described in Supplementary Table1.

| Sample type | Number of contrasts |
| --- | --- |
| A549 | 14 |
| AIRWAY_SMOOTH_MUSCLE_CELLS | 15 |
| ALVEOLAR_EPITHELIAL_CELLS | 8 |
| BAL | 237 |
| BLOOD | 1487 |
| BONE_MARROW | 3 |
| BRONCHIAL_EPITHELIAL_CELLS | 438 |
| CD4-T-CELLS | 78 |
| CD8-T-CELLS | 79 |
| DIAPHRAGM_MUSCLE | 6 |
| ENDOTHELIAL_CELLS | 5 |
| FIBROBLASTS | 134 |
| LUNG_BIOPSY | 1696 |
| LUNG_EXPLANT | 55 |
| MAMMARY_EPITHELIAL_CELLS | 26 |
| MONOCYTE | 74 |
| MYELOID_DENDRITIC_CELLS | 5 |
| THP-1 | 3 |
| TRACHEAL_EPITHELIAL_CELLS | 7 |
| U937 | 22 |
| VASTUS_LATERALIS_MUSCLE | 90 |

**Supplementary Table 5**. Number of contrasts per platform version, a platform type is also presented in an additional column.

| Platform version | Number of contrasts | Platform type |
| --- | --- | --- |
| [Glyco_v4_Hs] Custom Affymetrix Glyco v4 GeneChip | 5 | Affymetrix |
| [HG-Focus] Affymetrix Human HG-Focus Target Array | 17 | Affymetrix |
| [HG-U133_Plus_2] Affymetrix Human Genome U133 Plus 2.0 Array | 1110 | Affymetrix |
| [HG-U133A] Affymetrix Human Genome U133A Array | 260 | Affymetrix |
| [HG-U219] Affymetrix Human Genome U219 Array | 174 | Affymetrix |
| [HTA-2_0] Affymetrix Human Transcriptome Array 2.0 [transcript (gene) version] | 7 | Affymetrix |
| [Hu6800] Affymetrix Human Full Length HuGeneFL Array | 14 | Affymetrix |
| [HuEx-1_0-st] Affymetrix Human Exon 1.0 ST Array [transcript (gene) version] | 257 | Affymetrix |
| [HuGene-1_0-st] Affymetrix Human Gene 1.0 ST Array [transcript (gene) version] | 262 | Affymetrix |
| [HuGene-1_1-st] Affymetrix Human Gene 1.1 ST Array [transcript (gene) version] | 171 | Affymetrix |
| [HuGene-2_0-st] Affymetrix Human Gene 2.0 ST Array [transcript (gene) version] | 14 | Affymetrix |
| [HuGene10stv1_Hs_ENSG] Affymetrix GeneChip Human Gene 1.0 ST Array [Brainarray Version 11.0.1] | 276 | Affymetrix |
| Agilent-012097 Human 1A Microarray (V2) G4110B (Feature Number version) | 7 | Agilent |
| Agilent-014850 Whole Human Genome Microarray 4x44K G4112F (Feature Number version) | 457 | Agilent |
| Agilent-014850 Whole Human Genome Microarray 4x44K G4112F (Probe Name version) | 53 | Agilent |
| Agilent-028004 SurePrint G3 Human GE 8x60K Microarray (Feature Number version) | 436 | Agilent |
| Agilent-045997 Arraystar human lncRNA microarray V3 (Probe Name Version) | 1 | Agilent |
| Human 3.0 A1 | 42 | NA |
| Illumina HiSeq 2000 (Homo sapiens) | 216 | Illumina |
| Illumina HiSeq 2500 (Homo sapiens) | 5 | Illumina |
| Illumina HumanHT-12 V3.0 expression beadchip | 422 | Illumina |
| Illumina HumanHT-12 V4.0 expression beadchip | 211 | Illumina |
| Illumina HumanRef-8 v3.0 expression beadchip (Search Key version) | 52 | Illumina |
| Illumina HumanWG-6 v3.0 expression beadchip | 5 | Illumina |
| NimbleGen Homo sapiens HG18 expression array [100718_HG18_opt_expr_HX12] SEQ_ID condensed version | 4 | NA |
| Operon Homo sapiens 21K (Hs Operon Vw) | 3 | NA |
